# “The right people at the right time”: process evaluation of a novel allied health hospital in the home service for people with cancer

**DOI:** 10.1007/s00520-025-09694-1

**Published:** 2025-07-05

**Authors:** Ashlee Miller-Jenkins, Annie K. Lewis, Katherine Pryde, Amy M. Dennett

**Affiliations:** 1 Hospital in the Home, Eastern Health, Box Hill, VIC Australia; 2 Allied Health Clinical Research Office, Eastern Health, Box Hill, VIC Australia; 3 School of Allied Health, Human Services and Sport, La Trobe University, Bundoora, VIC Australia

**Keywords:** Allied health, Cancer, Rehabilitation, Implementation, Ambulatory, Home-based

## Abstract

**Purpose:**

To perform a process evaluation of the acceptability, adoption, costs, feasibility, safety, timeliness, and satisfaction of a novel allied health program in Hospital in the Home (HITH) cancer services.

**Method:**

A mixed-methods process evaluation using the proctor model for implementation was completed. Quantitative data from routinely collected service data, patient satisfaction surveys, and qualitative focus group data from cancer services’ staff over a 6-month period were analysed. Quantitative data were described, and qualitative data thematically analysed and mapped to seven key domains: acceptability, adoption, costs, feasibility, safety, timeliness, and satisfaction.

**Results:**

A total of 90 adults with cancer were referred to the allied health program in HITH cancer services, of which 69 (77%) participated. There were no major adverse events, and entry to the service was timely (median wait time: 5 days). Patients were satisfied with the service. Clinical staff reported the service “added value” by preventing hospital readmission and improving patient outcomes. The cost of the service was $518 AUD per patient contact (comparable inpatient stay: $5000 AUD).

**Conclusion:**

A timely home-based allied health cancer service can be achieved with adequate resources, communication, and collaboration. Future home-based models of allied health care for people with cancer should consider employing skilled staff and strategically aligning programs with health service priorities.

**Supplementary Information:**

The online version contains supplementary material available at 10.1007/s00520-025-09694-1.

## Introduction

People with cancer may experience symptoms that negatively impact quality of life, especially when undergoing anti-cancer therapies. Allied health professionals, such as occupational therapists and physiotherapists, can deliver evidence-based therapy to better manage symptoms and treatment side effects; mitigate adverse events like falls; promote independence and physical activity; and improve quality of life [1, 2]. In turn, functional interventions like exercise may prevent hospital admissions, cancer recurrence, and reduce the risk of premature death [3, 4].

Cancer treatment may follow a complex trajectory requiring a coordinated effort from multiple specialties to optimise an individual’s physical, psychosocial, cognitive, and emotional wellbeing [5, 6]. Although a multidisciplinary approach is widely accepted as gold standard for best-practice cancer care, current access to specialised allied health services is mainly offered in hospital-based settings during an inpatient admission, outpatient cardio-oncology rehabilitation, or geriatric assessment clinics [7]. Hospital-based models are resource-efficient and located where patients receive their medical care. However, there are barriers to reaching hospital-based allied health cancer services due to fatigue, competing medical demands, and travel time [8, 9].

Acute home-based care models have been developed to respond to the increasing demand on inpatient beds [10]. Home-based cancer services offer anti-cancer therapies in the home that would otherwise be delivered during hospital admission. These home-based cancer services are usually provided by doctors or nurses and rarely include allied health services. Emerging evidence demonstrates that home-based supportive care, such as exercise and psychotherapy, is beneficial for improving quality of life, especially among people with advanced cancer [11]. Multidimensional interventions improve patient care; however, the feasibility, including cost-effectiveness, of home-based programs is unclear [12]. General home-based rehabilitation services in Australia cost 28% less than an inpatient admission [13]. Evidence of these cost savings in a cancer setting is supported by a recent study of physiotherapy-led, home-based exercise for people with lung cancer, finding a net monetary benefit of AU$1508 favouring the intervention [14]. This suggests a need to better integrate ancillary services into home-based settings to ensure holistic, effective care during cancer treatment.

Process evaluations explore how interventions are implemented [15]. Studies related to cancer and allied health often evaluate feasibility or efficacy of interventions such as exercise. Few provide guidance on implementation to practice. A recent process evaluation of cancer telerehabilitation demonstrates how allied health interventions can be safely implemented in a pragmatic health service setting [16]. However, less is known about how home-based allied health services can be implemented for people undergoing cancer treatment. Without existing guidelines to support the implementation of home-based allied health services in cancer care [17], a process evaluation would provide practical guidance for service development. Therefore, the aim of this study is to perform a process evaluation exploring acceptability, adoption, feasibility, costs, safety, timeliness, and satisfaction of a novel allied health program in HITH cancer services.

## Methods

### Study design

**Fig. 1:**
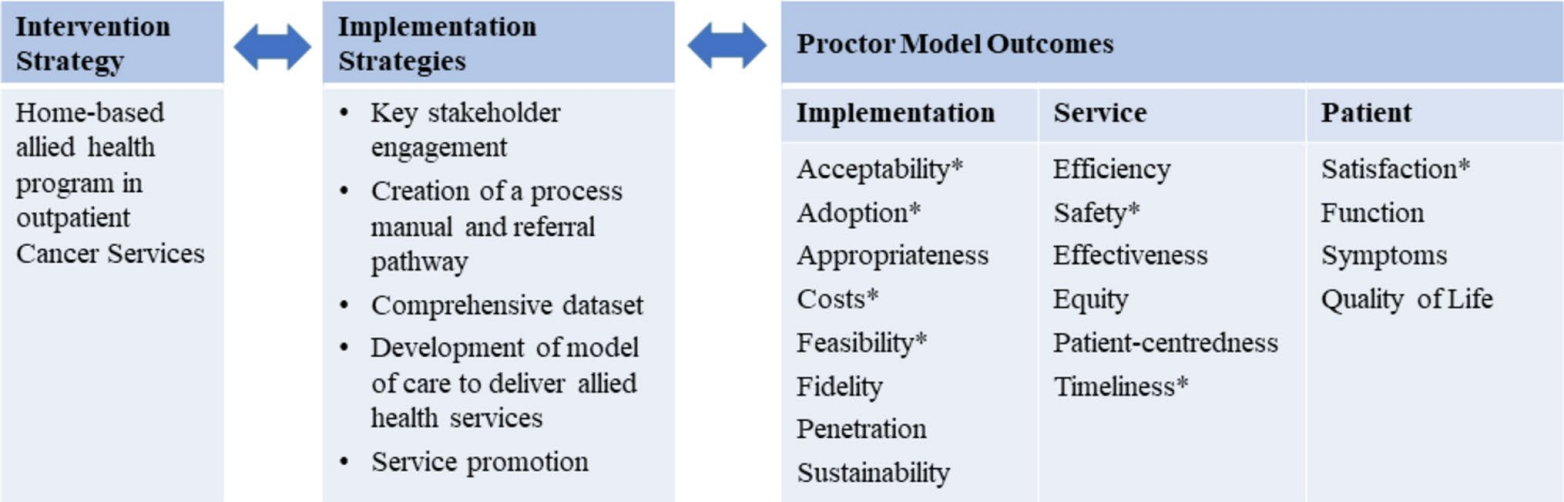
Illustration of HITH cancer services allied health program using the proctor model for implementation. *Outcomes included in this process evaluation

A process evaluation using mixed methods was completed to describe the development of a novel home-based, allied health model of care embedded in cancer services. This study was guided by the Proctor model for implementation evaluation framework (Fig. 1), comprising a taxonomy of three outcome categories (implementation, service, and patient), focusing on acceptability, adoption, costs, and feasibility; safety and timeliness; satisfaction. Ethical approval was obtained from the health service’s Human Research and Ethics Committee (LR22-070–90315). This study included quantitative and qualitative data reported according to the STrengthening the Reporting of OBservational Studies in Epidemiology (STROBE) statement [18] and template for intervention description and replication (TIDieR) checklist [19].

### Setting

The study was based in a large, publicly funded health service in metropolitan Melbourne, Australia, that admits approximately 3000 patients with cancer annually. Cancer services include a day oncology and medical unit (DOMU), which is supported by a Hospital in the Home (HITH) service where patients receive home-based anti-cancer treatments. HITH was previously a nurse-led service with hospital-based medical support if needed. Prior to this study, no dedicated home-based allied health services were provided to patients undergoing cancer treatment. A state-wide government funding initiative expanded home-based services in early 2022 [20], with a new allied health program developed to operate within HITH cancer services. Several implementation strategies aided adoption of the intervention [21]. These included: building a coalition with hospital clinical staff, managers, and administrators; conducting educational meetings (development and promotion of a referral pathway); developing educational materials (including a flyer added to a patient “welcome pack”); utilising an academic partnership and using an implementation advisor; developing and organising quality monitoring by creating a clinical database and providing feedback to clinical and leadership stakeholders; and obtaining and using consumer feedback to inform the intervention.

### Intervention

The intervention was the introduction of an allied health program to an established cancer HITH service (Table 1). Assessment and therapy were provided by an occupational therapist and/or physiotherapist at the location of the patient’s choice: face-to-face at home, in DOMU, or via telehealth. All patients were offered at least one initial assessment and a follow-up appointment. Therapy was individually tailored and may have included health coaching, supportive care, education, exercise, equipment prescription, or referral to community services. Frequency and duration of therapy were determined by clinicians.

**Table 1:** Intervention description using the Template for Intervention Description and Replication (TIDieR) checklist

INTERVENTION	
BRIEF NAME	· Home-based allied health program in HITH Cancer Services
WHY	· To reduce the need for psychosocial and/or functional hospital admissions and manage the coordination of community support services to improve quality of life and reduce symptom burden.
WHAT: MATERIALS AND PROCEDURES	· Occupational therapy and physiotherapy, individualised to patient need including: - Health coaching and supportive care - Written/digital education specific to cancer-related symptoms - Written or app-based (Physitrack), individualised home exercise program +/- exercise theraband - Trial/supply of aids and equipment to assist with functional mobility and activities of daily living - Referral(s) to community supportive care services.
WHO (PROVIDER)	· Occupational Therapist (0.4 EFT) and Physiotherapist (0.4 EFT) funded by the hospital. Staff have 23 years combined oncology experience and additional training in cancer care.
HOW	· Referral pathway and detailed program manual developed including service description, eligibility criteria and referral form with clinical indicators relevant for patient group. · Database constructed to capture data related to patient referrals (i.e. number, source, referral reason, demand, wait time), patient demographics, encounter location, attendance, nature and duration of allied health therapy, referrals made post allied health involvement. · Occupational therapy and physiotherapy appointment booked upon referral. · Comprehensive assessment conducted to determine patient’s need in the areas of symptoms and side effects of cancer and/or treatment, mobility, activities of daily living etc. · Therapeutic input provided based on patient goals and assessed risk.
WHERE	· Individual assessment and therapy occurred in patient’s choice of location (home/DOMU/telehealth)
WHEN / HOW MUCH	· Referrals triaged using Specific Timely Assessment and Triage (STAT) model principles, an evidence-based system used for access and triage in outpatient settings (22). · Urgent referrals seen within 7 days of referral. · Routine referrals contacted in chronological order of receipt, usually 7-14 days. · Frequency of patient contact determined by clinician and based on individual needs and assessment findings. - Initial contact: establish referral urgency, identify patient goals, schedule assessment (up to 30 minutes) - Assessment: Comprehensive functional assessment and intervention as indicated (up to 60 minutes) - Follow-up and discharge planning: up to 4 additional review appointments, community support referrals as required (up to 60 minutes)
TAILORING	· Individualised allied health therapy plan based on initial consultation and needs identified.
TRIAL FIDELITY	· Staff with a background in oncology who had prior formal training were employed by the hospital to provide the intervention. · Electronic records of the number of sessions provided. · Clinical supervision as per standard hospital policy.

### Participants

#### Patient participants

Study participants were patients referred to the allied health program between September 1, 2022, and March 1, 2023. Adult cancer survivors currently receiving, or within 12 months of receiving, outpatient anti-cancer treatment (curative or palliative intent) at the health service were eligible for the allied health cancer HITH program. People were considered ineligible if referred for issues not directly related to cancer, they had intensive rehabilitation needs, or lived in residential aged care.

For routinely collected data, individual patient consent was not sought, as clinical members of the research team had access to this data as per normal practice. Patients and caregivers who attended the allied health cancer HITH program also received a satisfaction survey. Consent for survey data was implied through its completion, which included a participant information sheet.

#### Staff participants

Allied health clinicians delivering the intervention, nurses and managers of HITH cancer services, and day oncology supporting staff were invited to participate in focus groups. Staff external to cancer services (e.g. general HITH staff) were excluded. Staff provided written informed consent prior to focus groups.

### Outcome measures

Data were collected from four sources (Table 2).

**Table 2:** Data sources for each outcome measure

	Key outcomes	Service Data	Patient and Caregiver Survey	Staff Focus Groups	Budget Data
Implementation	*Acceptability*		✓	✓	
	*Adoption*	✓		✓	
	*Costs*	✓		✓	✓
	*Feasibility*	✓	✓	✓	
Service	*Safety *	✓		✓	
	*Timeliness *	✓	✓	✓	
Patient	*Satisfaction *		✓	✓	

### Implementation outcomes

Acceptability, adoption, and feasibility were assessed by reviewing routinely collected data including referral, admission, encounter location, and attendance details. Analysis of therapy sessions documented in the medical record, including nature and duration of allied health intervention and ongoing referrals made, was also completed.

Program costs were estimated using calculation of staff salaries in line with relevant industrial agreements. Distance travelled for home visits was collected in a logbook. Equipment and travel costs were calculated using hospital financial records.

Patient demographics were collected from routine reports to describe the sample.

### Service outcomes

Timeliness was assessed by reviewing routinely collected wait-time data. Safety was assessed by reviewing adverse events from the medical record.

### Patient outcomes

Outcome measures to describe patient health status were routinely collected. These included baseline functional status (Australian-Modified Karnofsky Performance Status [AKPS]) [23]; cancer-related fatigue (Canadian Association of Psychosocial Oncology [CAPO] fatigue screen) [24]; falls risk (Falls Risk for Older People in the Community [FROP-Com]) [25].

#### Patient and caregiver survey

Acceptability and satisfaction of allied health service provision were measured via a purpose-made, consumer-reviewed online (QuestionPro, Dallas, TX) or paper survey (Appendix 1). The survey included a 5-point Likert scale with 12 statements about satisfaction with the allied health HITH service, rated from strongly disagree to strongly agree. Four open-ended questions were provided to allow additional information.

#### Staff focus groups

Two 1-h focus groups were completed to allow participants the opportunity to discuss their experience relating to program implementation. Leaders and clinicians were interviewed separately to allow participants the ability to freely express their views. Focus groups were facilitated by an experienced PhD-qualified researcher (AD) with a background in cancer physiotherapy, not involved with the implementation or delivery of the allied health HITH service. The interview schedule focused on acceptability, adoption, costs, feasibility, safety, and timeliness (Appendix 2). Participants received the interview schedule prior to the session to allow them to prepare.

## Data analysis

Patient participant characteristics, acceptance, safety, costs, and satisfaction associated with the allied health HITH service were analysed descriptively. Content of open-ended survey comments was coded and grouped deductively into categories of the Proctor Model for Implementation Framework. Participant interviews were audio-recorded and transcribed verbatim. Transcripts were de-identified and assigned an identification number. Transcripts were independently coded inductively line-by-line by three researchers (AMJ, AD, and AL) using open coding (i.e. the codes emerged from the data). AL is a PhD-qualified health services researcher and occupational therapist with managerial experience employed by the health service. AMJ is an occupational therapist and novice clinician researcher who implemented the allied health HITH service. Codes were categorised and discussed until consensus was reached on main themes. Themes were then mapped deductively onto the Proctor Model.

## Results

**Fig. 2:**
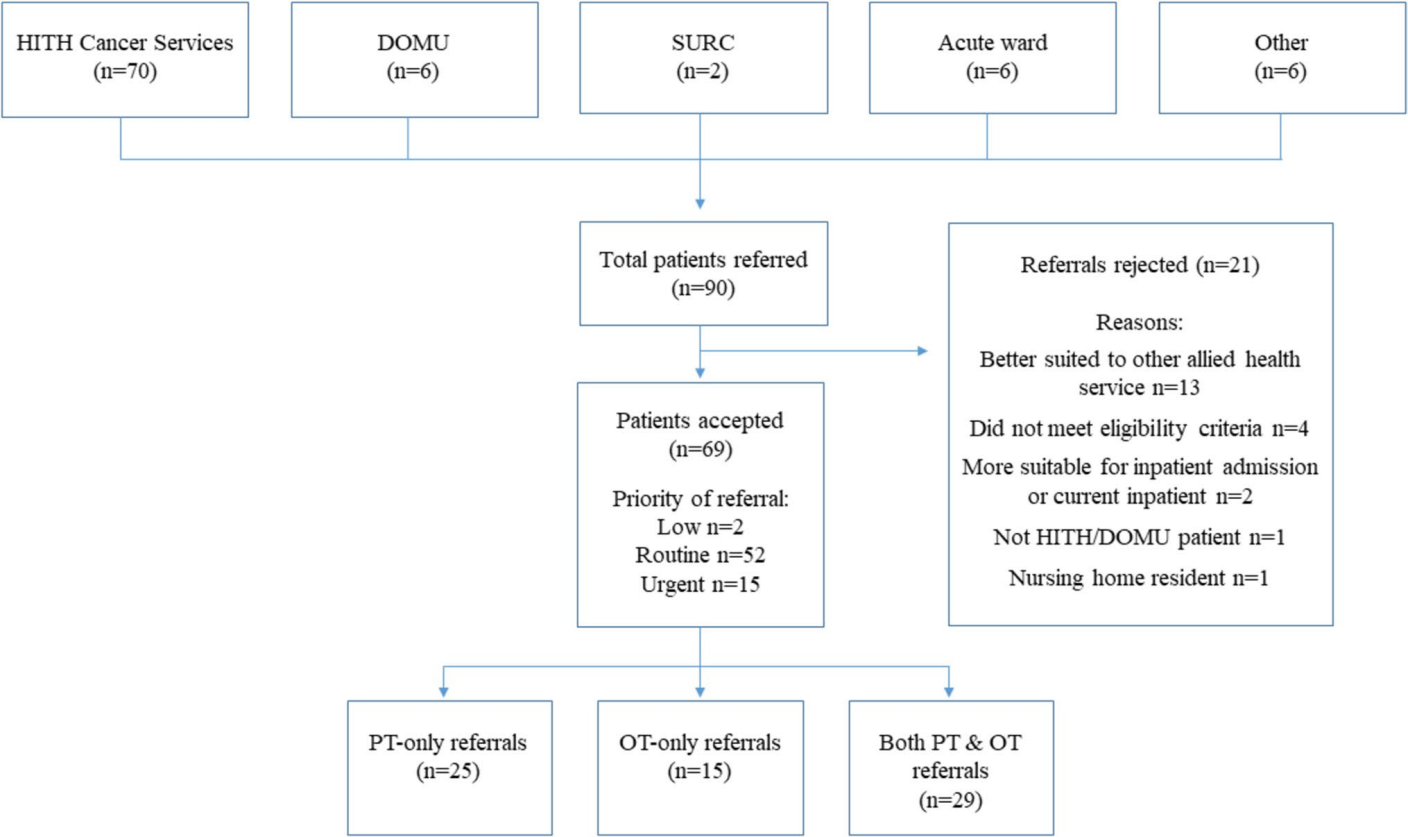
Flow of referrals

During the 6-month data collection period, 90 people were referred to the allied health HITH service, of which 69 (77%) were eligible and accepted (Fig. 2). Most referrals (*n* = 64, 93%) were generated by nurses. The most common reasons for referral to occupational therapy were home safety assessment (*n* = 26) and functional independence changes (*n* = 23). The most frequent reasons for referral to physiotherapy were falls risk (*n* = 27), deconditioning (*n* = 22), and recent change in mobility (*n* = 20).

Nine staff (including two program leaders, one medical oncologist, three associate nurse unit managers, one clinical nurse consultant, and two allied health clinicians) participated in focus groups.

Most patient participants (*n* = 39, 56%) were male, with a mean age of 71 years (SD 13, range 34 to 94) (Table 3). Most had haematological malignancies (*n* = 36, 52%). Over half of participants had advanced cancer (*n* = 47, 68%). On average, patient participants had a baseline performance score of 70, indicating an inability to carry on usual work due to their disease. Over half reported moderate (*n* = 34, 49%) or severe (*n* = 16, 33%) levels of fatigue, and many were identified as high risk of falls (*n* = 28, 41%).

**Table 3:** Baseline characteristics (patient participants)

Characteristics	*n* = 69
Mean age, years (SD), [range]	71 (13) [34–94]
Age group, *n (%)*	
18–64	18 (26)
65 +	51 (74)
Gender, female, *n (%)*	31 (44)
Cancer type, *n (%)*	
Colorectal	7 (10)
Breast	7 (10)
Lung	8 (12)
Skin	1 (1)
Haematological malignancies	39 (57)
Head and neck	1 (1)
Upper GI	6 (9)
Cancer stage, *n (%)*	
Early	3 (4)
Advanced	47 (68)
Not known	19 (28)
AKPS performance status, *n (%)*	
0–40	1 (1)
50	7 (10)
60	19 (28)
70	19 (28)
80	10 (14)
90	2 (3)
Not known	11 (16)
FROP-COM, *n (%)*	
Low risk (0–3)	32 (46)
High risk (4–9)	28 (41)
Not known	9 (13)
CAPO fatigue, *n (%)*	
No/mild	8 (12)
Moderate	34 (49)
Severe	16 (23)
Not known	11 (16)

^a^The sum of percentages may not reach 100 because of rounding

*Abbreviations:* *GI *gastrointestinal, *CNS *central nervous system, *AKPS* Australian Modified Karnovsky Performance Scale, *FROP-COM *Falls Risk for Older People in the Community assessment, *CAPO *Canadian Association of Psychosocial Oncology

## Implementation outcomes

### Acceptability

**Fig. 3:**
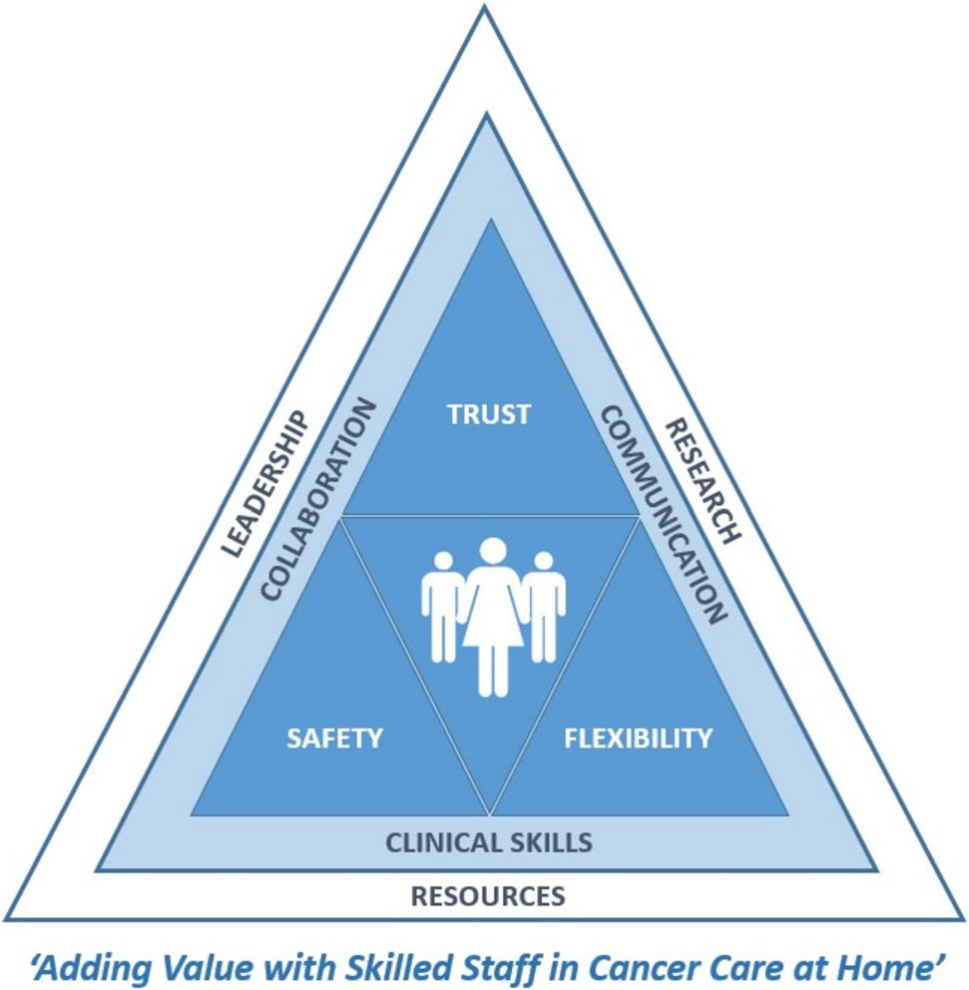
Interpretive synthesis illustrating the implementation of a patient centred allied health HITH service

Overall, the allied health cancer HITH service was well accepted by staff, as reflected by the high number of referrals and positive comments made by clinical and leadership staff (Appendix 3). The program was described as “*adding value*” to care already provided by the organisation’s existing cancer services (Fig. 3).

“So valuable, approachable, open communication, they’ve been a breath of fresh air.” [Staff Participant 3, Nurse]

Acceptability of the service was positively influenced by discipline-specific clinical skills and experience, and interpersonal skills resulting in trust and strong communication within the team.

“Their communication to us, backwards and forwards is outstanding!” [Staff Participant 3, Nurse]

## Adoption

A total of 191 patient contacts were recorded (OT = 82, PT = 109). Most of these were conducted at home (167/191, 87%) with a small number of contacts occurring in the hospital/clinic setting (24/191, 13%). Physiotherapy was mostly delivered in person (*n* = 60, 55%) and occupational therapy delivered via telephone (*n* = 51, 62%). Staff were impressed with how well allied health integrated into the existing HITH cancer services team. This was attributed to the collaborative, flexible, and pro-active approach of allied health staff.

“Nothing’s too difficult, that can-do attitude. It’s wonderful.” [Staff Participant 1, Leadership]

Leaders expressed a desire for this allied health model to be adopted elsewhere, given their perceived value and the organisation’s strategic direction. They also described how other health services had enquired about adopting this program in their organisations.

“I’m very much hoping that we will get ongoing funding… it’s one of our strategic directions, [for] more care at home.” [Staff Participant 2, Leadership]

## Costs

There was no out-of-pocket cost to patients receiving allied health intervention, as per public hospital funding in Australia. The total service cost, including set-up, was $98,867 AUD. The cost of the service was $518 AUD per patient contact. This included telecommunications equipment (i.e. laptops and mobile phones) and a fleet vehicle. The primary resource cost was clinical staff salaries (Appendix 4). Total distance travelled for home visits between September 2022 and March 2023 was 1873 km, which totalled $1592 AUD.

“It’s very much like what you would need on a ward…the added component of cars, phones, laptops, it’s like having a little mini hospital in the car.” [Staff Participant 2, Leadership]

Of the 69 patient participants, 27 were provided with aids and equipment (e.g. shower stools, toilet aids, gait aids, bed supports). Equipment was funded by HITH for 30 days, as per hospital policy. A total of 50 equipment items were prescribed, costing $2164 AUD.

Proactive interventions that enable independent living were perceived to help patients avoid hospital admission, and the program’s responsiveness meant staff sensed overall cost savings to the health service.

“You’re not going to have to go to emergency…get admitted to hospital, we can keep you at home.” [Staff Participant 4, Oncologist]

## Feasibility

The allied health HITH program was perceived as feasible, facilitated by skilled, collaborative staff, and having dedicated resources and protected time for program set-up. Strong, flexible leadership, including managers and clinician researchers, supported the service evaluation and adapting to service demands.

“I think we were very lucky, because we had the right people at the right time fitting in...” [Staff Participant 1, Leadership]

On average, two (range 1–5) therapy sessions were provided. The length of admission ranged from 0–56 days for occupational therapy, and 0–41 days for physiotherapy. Just 11 (6%) sessions were not attended. For many patients, allied health staff identified the need for ongoing support at discharge and made 70 individual referrals to community services (Appendix 5).

## Service outcomes

### Safety

The most common occupational therapy interventions were falls prevention strategies (23/44, 52%), equipment prescription (21/44, 48%), and energy conservation techniques (18/44, 41%). Other interventions included home modifications, pressure care, and upper limb therapy. Physical activity education (49/54, 91%) and home exercise programs (39/54, 72%) were common for patients following physiotherapy assessment. Other therapy interventions included fatigue management education, falls prevention, and health coaching.

There were no adverse events related to allied health intervention recorded. All staff perceived the intervention as safe in what they believed to be a vulnerable cohort of patients. Having experienced oncology staff was described as a key factor.

“How wonderful it is to have experienced allied health staff… Not really that we’re special or unique, it’s just clinically safer.” [Staff Participant 5, Nurse]

## Timeliness

Demand was proactively monitored, with protected new appointments in clinician schedules balanced with referral numbers to ensure timely access [22]. The median wait time from referral to first contact by the service was 5 days (range 0–16).

“I think they need to be responsive, as soon as possible, because what it would mean…[nursing] staff ask for readmission back into hospital, which could be prevented.” [Staff Participant 3, Nurse]

Patients of the service recognised the timeliness of the program, with most survey respondents (21/22; 95%) agreeing “*allied health staff made contact within a reasonable timeframe*”. Staff participants also described the allied health HITH service as timely, which was credited to strong communication by the allied health team and easy referral processes.

## Patient outcomes

### Satisfaction

Surveys were returned by 22 participants (16 patients; 6 carers). All survey respondents agreed they were “*very satisfied with the allied health services provided*”, and all recognised the importance of having access to allied health interventions in their own home. Participants commented on the value of having knowledgeable, experienced staff in relation to cancer. Positive feedback was provided regarding exercise programs, equipment prescription, and coordination of community support referrals (Appendix 6).

“Home visits have been a blessing at a time when it was very difficult to leave home.” [Participant 10, Patient]

Few suggestions were offered on how to improve the program, other than many patient participants identifying medical home visits as highly desirable if there was further growth of the program (14/22, 63%).

## Interpretive synthesis

The overarching theme arising from the data was skilled allied health staff in home-based cancer care added value (Fig. 3). This was achieved by putting patients at the centre of all encounters, enhanced by highly developed communication, discipline-specific clinical skills, and collaboration. Patients and the broader cancer services team trusted allied health professionals to provide safe and flexible care that optimised outcomes. This was achieved within the context of having sufficient resources to build the service and evaluate its success with support from leadership staff.

## Discussion

This new home-based allied health program provided people with cancer timely allied health assessment and treatment to improve quality of life while preventing avoidable hospital admissions. A strength of this program was that it was integrated with the existing home-visiting cancer nursing service, with additional dedicated funding to formally develop and evaluate the model. This program demonstrated clear demand for home-based allied health cancer services and was safe and well accepted by staff and patients, with low non-attendance rates and desire for adoption of the model by other services. This study adds to existing literature by describing how a home-based allied health program can be implemented into a busy public hospital cancer service and inform future implementation of similar evidence-based services in other settings.

This home-based model of care appeared to increase access to allied health professionals for people with cancer. Access to allied health interventions is generally scarce [26], and often only exists in inpatient services [27] or outpatient exercise rehabilitation programs [26], with only half of cancer centres providing an established pathway to allied health [28]. This program adopting a home-based model of care presents a potential solution to this issue. Flexibility in location and mode of delivery are likely to have reduced patient-related barriers to attendance, contributing to favourable patient participation.

There was strong organisational support for this program. Common organisational barriers to implementation of allied health services such as lack of funding, skilled staff, and leadership support [29] were overcome through dedicated funding, strong leadership buy-in, and integration with an existing service. Moreover, the program aligned with the strategic direction of the health service to provide more care at home and highlighted the importance of adequate resources, communication, and collaboration to achieve successful implementation. Future allied health cancer programs should consider how they fit within broader organisational structures to provide benefits to not only patients but also the health service.

A key strength of this program was the perception that timely allied health intervention reduces costs to the health service by preventing unnecessary hospital admission. The cost of home care is approximately 10% of inpatient care at this health service [30]. The cost of staying in a hospital bed overnight or receiving daytime cancer services in Australia is approximately $5000 AUD [30]. In this study, the cost was $518 per patient contact. Additional cost savings may be further realised over time as initial set-up costs for cars and equipment are absorbed. Preventable hospital admissions due to modifiable issues such as falls and pressure injuries present high costs to health services [32]. This is a significant issue, given people receiving taxane-based chemotherapy have approximately 40% greater risk of falling than those who do not [31], and older cancer survivors have a 16% greater risk than older people without cancer [32]. Interventions such as exercise and rehabilitation may reduce hospital readmission during cancer treatment [4, 33], therefore home-based allied healthcare has the potential to provide significant cost savings to health services [31].

Our findings indicate the introduced allied health program provided “patient-centred care” [34], and this study highlighted the importance of specialised allied health staff providing interventions to people with cancer. The importance of specialised knowledge and skills in cancer rehabilitation, including knowledge of interventions to address common cancer symptoms, strong communication skills, and awareness of community supports, ensures safety and trust in cancer service delivery [9, 35, 36]. Currently, few formal training opportunities exist to upskill cancer care clinicians [37–39]. Therefore, professional development in core clinical skills related to cancer care such as cancer-related fatigue management, physical activity education, and supportive care should be available to allied health staff to develop the oncology workforce and provide evidence-based, effective cancer services.

## Strengths and limitations

To our knowledge, this is the first study to conduct a process evaluation with a novel, home-based allied health program in cancer services. This intervention was reported using the TIDieR guidelines [19], which will assist with translation of findings in other cancer settings. It was conducted in a pragmatic health service setting to improve generalisability. Feedback was also obtained from one-third of service users, which aligned largely with other qualitative studies of cancer allied health services, increasing our confidence in the data [16, 40].

Limitations of this study include the inclusion of just one health service and only access to two allied health disciplines: occupational therapy and physiotherapy. Differing outcomes may be observed in other disciplines. However, these disciplines are supported by high levels of evidence and are proportional to the size of the allied health workforce at this health service. Data related to hospital readmission and cost savings were subjective, and a full cost-effectiveness study was not completed, as the aim of this study was to describe implementation rather than cost. However, our cost data may provide an initial understanding of the potential for cost benefits.

## Conclusions

This study demonstrated integration of allied health into a home-based cancer services model is feasible and acceptable to patients, caregivers, and referrers, with few challenges identified. Adequate resources, communication, and collaboration facilitated successful implementation. Future home-based models of allied health care for people with cancer should consider employing skilled allied health staff and strategically align programs with health service priorities to improve access to supportive care.

## Supplementary Information

Below is the link to the electronic supplementary material.

ESM 1 (DOCX 34.0 KB )

ESM 2 (DOCX 34.0 KB)

ESM 3 (DOCX 42.5 KB)

ESM 4 (DOCX 32.1 KB)

ESM 5 (DOCX 62.1 KB)

ESM 6 (DOCX 30.4 KB)

## Data Availability

Data is available as supplementary material.
