# Supplementary material for: “The right people at the right time”: process evaluation of a novel allied health hospital in the home service for people with cancer
Source: Support Care Cancer. 2025 Jul 5;33(7):658. doi: 10.1007/s00520-025-09694-1 (PMC12228668; doi:10.1007/s00520-025-09694-1)
Supplement: Supplementary file 1 — (DOCX 34.0 KB ) [file 520_2025_9694_MOESM1_ESM.docx]

**Appendix 1: Participant Satisfaction Survey**

**Please answer the following questions in relation to your allied health experience with Hospital in the Home (HITH) Cancer Services** **at Eastern Health.**

**Allied Health refers to Occupational Therapy and/or Physiotherapy.*

**I am a:**

- Patient
- Carer / Family / Friend / Loved one
- Other (please describe):

**Which allied health services did you receive?** (You can tick more than one answer)

- Occupational Therapy
- Physiotherapy

**How was your allied health session(s) delivered?** (You can tick more than one answer)

- Face-to-face in your home
- Face-to-face in the Day Oncology Medical Unit (DOMU)
- Phone
- Videoconference

**Were you satisfied with the location of the allied health session?**

- Yes
- No
- I would have preferred:

|  | **Strongly Disagree** | **Disagree** | **Neither Agree nor Disagree** | **Agree** | **Strongly Agree** |
| --- | --- | --- | --- | --- | --- |
| 1. It is important for people with cancer to have access to allied health services at home. | 1 | 2 | 3 | 4 | 5 |
| 1. Allied health staff made contact within a reasonable timeframe. | 1 | 2 | 3 | 4 | 5 |
| 1. Allied health staff were on time with their visits. | 1 | 2 | 3 | 4 | 5 |
| 1. Allied health staff responded to all of my questions and concerns. | 1 | 2 | 3 | 4 | 5 |
| 1. Allied health staff were friendly, respectful and non-judgemental. | 1 | 2 | 3 | 4 | 5 |
| 1. Allied health staff provided education and support to improve home safety. | 1 | 2 | 3 | 4 | 5 |
| 1. Allied health staff were knowledgeable about the impacts cancer can have on someone’s life. | 1 | 2 | 3 | 4 | 5 |
| 1. Allied health staff communicated well with me. | 1 | 2 | 3 | 4 | 5 |
| 1. Allied health staff completed all follow-up (i.e. referrals) as discussed during our session. | 1 | 2 | 3 | 4 | 5 |
| 1. I feel that my overall health and wellbeing has improved due to the allied health services provided. | 1 | 2 | 3 | 4 | 5 |
| 1. Allied health services helped keep me/my loved one at home and out of hospital. | 1 | 2 | 3 | 4 | 5 |
| 1. Overall, I am very satisfied with the allied health services provided. | 1 | 2 | 3 | 4 | 5 |

**What aspects of the allied health at home program did you find most helpful?**

|  |
| --- |

**What were the challenges (if any) that you faced with the allied health at home program?**

|  |
| --- |

**How could we make the allied health at home program better?**

|  |
| --- |

**What other services would you find helpful at home?** (You can tick more than one answer)

- Social Work
- Dietetics
- Speech Pathology
- Psychology
- Medical – home-visits
- Other (please list):

**Additional comments:**

|  |
| --- |

**Thank You!**

If you would like to share your experience of Australian Health Services, tell us what was good and what could be improved, say thanks or call for change, please visit [www.careopinion.org.au](http://www.careopinion.org.au)
