# Supplementary material for: “The right people at the right time”: process evaluation of a novel allied health hospital in the home service for people with cancer
Source: Support Care Cancer. 2025 Jul 5;33(7):658. doi: 10.1007/s00520-025-09694-1 (PMC12228668; doi:10.1007/s00520-025-09694-1)
Supplement: Supplementary file 2 — (DOCX 34.0 KB) [file 520_2025_9694_MOESM2_ESM.docx]

**Appendix 2: Interview Schedule for Clinicians**

| Proctor Domain | Evaluation Question | Interview Question |
| --- | --- | --- |
| Acceptability | Is the intervention accepted by patients and clinicians? | Prior to 2022, HITH Cancer Services was nurse-led with no employed allied health staff. What has been your experience of having allied health delivered within your service compared to the previous model of care?  What is your current understanding of what allied health services can offer patients? Has your level of understanding changed since the HITH allied health service started?  How willing were you to refer patients to allied health before HITH had its own allied health team? Has this changed since the HITH allied health service started in 2022?  How willing were patients to accept your recommendation to involve allied health?  What have been the benefits vs challenges of having allied health join HITH Cancer Services? |
| Adoption | What is the likelihood of clinicians choosing to adopt the intervention beyond the current funding-related time period? | Given the choice, would you continue to adopt a model of care inclusive of allied health going forward? Why/why not?  What do you think about this model being adopted by other health services?  What support from key stakeholders is required for this model to be adopted long-term? |
| Feasibility | Can the intervention be successfully delivered in this setting? | What factors supported the delivery of allied health services in this setting (e.g. supportive care screening)?  What were the positives and challenges? |
| Safety | Is the intervention safe? | How safe was allied health intervention? |
| Timeliness | Is the intervention provided within a timeframe acceptable to patients, caregivers and clinicians? | How do you feel about the allied health clinicians’ response time to referrals?  How do you feel about the timeliness of allied health interventions? |
| Satisfaction | Are staff, patients and caregivers satisfied with the intervention? | Overall, how satisfied are you with the new allied health service?  What feedback (if any) have you received from patients who received HITH allied health intervention? |

**Interview Schedule for Managers**

| Proctor Domain | Evaluation Question | Interview Question |
| --- | --- | --- |
| Acceptability | Is the intervention accepted by patients and clinicians? | What was your experience of having this intervention delivered within your service?  What feedback did you receive from staff or patients related to the intervention?  How appropriate was it to integrate allied health clinicians into the HITH Cancer Services team? |
| Adoption | What is the likelihood of management choosing to adopt the intervention beyond the current funding-related time period? | Given the choice, would you continue to adopt a model of care inclusive of allied health going forward? Why/why not?  Could you see this model of care being applied to other settings in future?  What resources would be required to adopt this model long-term? |
| Feasibility | Can the intervention be successfully delivered in this setting? | What factors supported the delivery of allied health services in this setting?  Overall, how successful was the allied health model of care? What were the barriers and facilitators? |
| Costs | What are the costs of implementing Allied Health in HITH Cancer Services? | What costs are involved with including allied health in HITH Cancer Services?  How well was the department able to support costs involved in delivering allied health in HITH cancer services? |
| Safety | Is the intervention safe? | How safe was allied health intervention? Prompt: Have there been any VHIMS including allied health services? |
| Timeliness | Is the intervention provided within a timeframe acceptable to patients, caregivers and clinicians? | What feedback about the timeliness of response to allied health referrals have you received? |
| Satisfaction | Are staff, patients and caregivers satisfied with the intervention? | Overall, how satisfied are you with the new allied health service?  Do you think patients, caregivers and staff are satisfied with the service? |
