# Supplementary material for: “The right people at the right time”: process evaluation of a novel allied health hospital in the home service for people with cancer
Source: Support Care Cancer. 2025 Jul 5;33(7):658. doi: 10.1007/s00520-025-09694-1 (PMC12228668; doi:10.1007/s00520-025-09694-1)
Supplement: Supplementary file 3 — (DOCX 42.5 KB) [file 520_2025_9694_MOESM3_ESM.docx]

**Appendix 3: Selected Staff Interview Quotes Mapped to Proctor Model for Implementation**

| Proctor Outcome | Sub-theme | Quote |
| --- | --- | --- |
| Acceptability | ***Patient First*** | - *“It's just such a wonderful, wonderful service that they offer, and I just can't speak highly enough of them.”* [Participant 1, Leadership] - *“I don't know where we would be without them to be honest.. And patients are so grateful.”* [Participant 3, Nurse] |
|  | ***Trust*** | - *“There is always that touch base, it might not be the physical assessment, but there's that touch base telehealth assessment, which is often comforting to the patient to know that there is an allied health team on board.”* [Participant 3, Nurse] |
|  | ***Safety*** | - *“I did have one patient when a nurse came back and said, Oh my gosh, I think this person is a really high falls risk. And it was a Thursday. You know, [physio] wasn't going to be back until the following Tuesday, but then [OT] could go out and at least we sort of tried to work a little bit interdisciplinary in that way to minimise the clinical risk for the patient as well if we can, or set them up with a bit of equipment or whatever it might be put something in to try to minimise their risk before we can get the proper assessment.”* [Participant 9, Allied Health Clinician] |
|  | ***Flexibility*** | - *“So valuable, so approachable, open communication, they've been a breath of fresh air.”* [Participant 3, Nurse] - *“Easy to get along with… they're professional and approachable… they know what they’re doing.”* [Participant 7, Nurse] |
|  | ***Clinical Skills*** | - *“I will be happy to refer my parents to my allied health team... They're very good at what they do. And they’re caring.”* [Participant 6, Nurse] |
|  | ***Collaboration*** | - *“Talk about it, discuss it together. So it's very complimentary. I think we sort of work together very well, as a team.”* [Participant 6, Nurse] - *“I'm incredibly proud of what our allied health team and what we've achieved, what they have achieved.”* [Participant 2, Leadership] - *“So very proud of how they've just made our team we're good but now is super good!“* [Participant 6, Nurse] |
|  | ***Communication*** | - *“Communication is very easy. And [OT] and the team know exactly what you're asking them and are very proactive at trying to sort out a plan or if they can't work out a plan, you know, work out another solution.”* [Participant 4, Oncologist] - *“Their communication to us, backwards and forwards is outstanding. They're just right on the ball, it's and the ease of doing it.”* [Participant 3, Nurse] - *“They have consistent with that contact that communication and I think that's what's aided in the satisfaction of the patients so the patients actually know what's going on.”* [Participant 1, Leadership] |
| Adoption | ***Trust*** | - *“They're the specialists, we trusted their ability to work through, you know, what are their referral indicators, we trust their judgement.”* [Participant 2, Leadership] - *“If someone's a little bit out of the box, we trusted their clinical judgement that that person needed to be seen.”* [Participant 2, Leadership] |
|  | ***Safety*** | - *“They've got an amazing handbook that, you know, it's not just for succession planning, but it's also to be very clear to everybody who comes into that team. This is how we manage the patients.”* [Participant 1, Leadership] |
|  | ***Flexibility*** | - *“Nothing's too difficult, that can-do attitude. It's sort of like, it's, it's wonderful.”* [Participant 1, Leadership] |
|  | ***Collaboration*** | - *“It wouldn't have worked if we didn't have an amazing, really strong, welcoming nursing team as well. Our nursing team have been amazing and accepting them and bringing them into the fold, as well.”* [Participant 2, Leadership] - *“The multidisciplinary team.. we don't operate in silos, I feel like it's really quite open… and expertise is respected.”* [Participant 5, Nurse] |
|  | ***Communication*** | - *“That's what's been part of the success of having that team… they were just so robust in setting up their, you know, making their guides, making their plans, and just their communication.”* [Participant 1, Leadership] |
|  | ***Leadership*** | - *“I have had extensive experience in developing new models of care. And I've actually learned over my years of management, they actually needed to be part of the development and to really run, you know, like drive it. And to me… the results have been really great.”* [Participant 2, Leadership] - *“…allowing the team to develop, you know, like, within reason to develop, organically…. we let them organically develop without a rigid set of rules.”* [Participant 2, Leadership] - *“I'm very much hoping that we will get ongoing better at home funding … particularly for our organisation, it's one of our strategic directions, its more care at home. So I've also just put in a business case for HITH General to have developed an allied health team, similar to what we've done in oncology.”* [Participant 2, Leadership] |
| Costs | ***Patient First*** | - *“If you can write a business case to set up an allied health team in your HITH oncology, or you're at home oncology services, absolutely do it because you won't look back.”* [Participant 1, Leadership] |
|  | ***Safety*** | - *“I think, from a medical side of things, I think the main value they add is reducing hospitalization.”* [Participant 4, Oncologist] |
|  | ***Flexibility*** | - *“To be able to get a relatively rapid Allied Health Review in their home to try to work out a plan rather than, you know, I suspect all these patients used to previously end up going into ED.”* [Participant 4, Oncologist] |
|  | ***Clinical Skills*** | - *“You're not going to have to go to emergency, you're not going to have to get admitted to hospital, we can keep you at home. And I think that's wonderful.”* [Participant 2, Leadership] |
|  | ***Communication*** | - *“They add so much value to the team and to the HITH oncology patients.”* [Participant 1, Leadership] |
|  | ***Resources*** | - *“It's also the cost of the equipment and the ongoing hiring cost, while the patient's under our care as well, which needs to be factored in. … It's very much like what you would need on a ward. But you know, the added component of cars, and phones, laptops, and it's like having a little mini hospital in the car.”* [Participant 2, Leadership] |
| Feasibility | ***Patient First*** | - *“The other component is the ongoing care. So, oncology HITH pulls out. But our patients are actually getting referred on for ongoing care provision through other services. … improving their quality of life, decreasing the risk of readmissions… optimising their goals. That journey for that patient … we know [they are] going to get the appropriate care once they leave our service as well”.* [Participant 2, Leadership] |
|  | ***Safety*** | - *“I think that times there's a real lack of clinical appreciation of haem oncology patients. That's why I said before, how wonderful it is to have experienced allied health staff who have that experience. Not really that we’re special or unique, it's just that it's clinically safer, it feels to me every time.”* [Participant 5, Nurse] |
|  | ***Flexibility*** | - *“We've spent nearly two and a half years trying to recruit a social worker. So that was a significant barrier to us. But it also provided opportunity as well … we were able to pivot and change the EFT profile to include a dietitian, because we, we actually realised what we needed, that was a fairly high need … was nutritional support. So whilst it was a barrier… it actually gave us the opportunity to rethink our model as well, which [was] a great benefit for our patients.”* [Participant 2, Leadership] |
|  | ***Clinical Skills*** | - *“The calibre of Allied Health staff has made a difference to the experienced oncology practitioners, so they know about chemotherapy and about the side effects of fatigue in the fact there’s cycles, that's really been a benefit as well.”* [Participant 5, Nurse] - *“I think we were very lucky, because we had the right people at the right time fitting in, you know, like said, just so well into the team, that it just worked.“* [Participant 1, Leadership] |
|  | ***Collaboration*** | - *“I can't believe how seamlessly they have actually fit into the team and how passionate and effective they have actually been.”* [Participant 1, Leadership] - *“As I said, it's seamless, it's like, the nurses will identify it, they will go and have a conversation to [OT] or to [Physio]. And this is initially but as the chains grown now. And they would just it would just be like, Oh, now we have someone to just go and have a quick discussion. What can I do? What can you do? And it's done. And nothing's too hard for them. And that's what I mean about the value that they've added.”* [Participant 1, Leadership] |
| Safety | ***Safety*** | - *“Avoiding admissions, keeping them safe, and as well as possibly nursing home, living independent lives.”* [Participant 5, Nurse] - *“It's been a very safe service. I definitely haven't had any feedback of any issues with safety.”* [Participant 4, Oncologist] - *“I think it's absolutely safe. … I haven't seen any VHIMs, related to any of the care provision they've had.”* [Participant 2, Leadership] |
|  | ***Clinical Skills*** | - *“I think that times there's a real lack of clinical appreciation of haem oncology patients. That's why I said before, how wonderful it is to have experienced allied health staff who have that experience. Not really that we’re special or unique, it's just that it's clinically safer, it feels to me every time.”* [Participant 5, Nurse |
|  | ***Communication*** | - *“We've formalised a sort of escalation pathway for if, you know, allied health arrived, and someone's unwell, you know what steps need to be done, and that was sort of in collaboration with medical and nursing team.”* [Participant 4, Oncologist] - *“If they get to a person's home, and they feel unsafe, or that the place is unsafe, or there's any issues with the deterioration that will escalate that in a very timely manner. And there's always the feedback and the communication, post the visit. So we're having those group discussions, conversations, that we can actually knock out any other further issues regarding safety.”* [Participant 3, Nurse] |
| Timeliness | ***Patient First*** | - *“I think it prevents that readmission, but also supports the patient in like, a timely manner to without them kind of missing out...”* [Participant 8, Allied Health Clinician] - *“We've now got a direct referral part of the team, allied health on tap. It's wonderful. So our patients are getting prompt allied health review and care intervention straightaway.”* [Participant 5, Nurse] |
|  | ***Flexibility*** | - *“They're not going to leave someone who needs something immediately for two or three weeks... So I think, you know, they have that ability to respond. They have a triage ability.”* [Participant 1, Leadership] - *“They are able to identify who needs what, sooner than later and respond accordingly. And I think that's, that's the beauty of this team that they are able to manage that very appropriately.”* [Participant 1, Leadership] |
|  | ***Clinical Skills*** | - *“I think it's absolutely they need to be responsive, as soon as possible, because what it would mean is us first nursing staff is to possibly speak to the doctors to ask for readmission back into hospital, which could be prevented if these clients have these, their appropriate Allied Health Services tapped in within.”* [Participant 3, Nurse] - *“It might not be the actual home assessment to the home in a physical presence, but it's that phone consult, which actually also makes that difference.”* [Participant 3, Nurse] - *“I think they are a very responsive team, but I think they're also they, their assessment skills are amazing, and they are able to react, depending on the needs of the patient.”* [Participant 1, Leadership] |
|  | ***Collaboration*** | - *“So straight away, you can just email the girls and get Yep, I'll come out, I'll see them. Or if we brought a patient in for review. They're straight onto it. So it's so helpful.”* [Participant 7, Nurse] |
|  | ***Communication*** | - *“As they've been so well integrated into the team, their referrals have increased ... I know [physio] has a bit of a waitlist, but I think it's their communication, their timely communication, it's made it so easy. And particularly with the referrer as well as the patients you know.”* [Participant 1, Leadership] |
