# Supplementary material for: “The right people at the right time”: process evaluation of a novel allied health hospital in the home service for people with cancer
Source: Support Care Cancer. 2025 Jul 5;33(7):658. doi: 10.1007/s00520-025-09694-1 (PMC12228668; doi:10.1007/s00520-025-09694-1)
Supplement: Supplementary file 4 — (DOCX 32.1 KB) [file 520_2025_9694_MOESM4_ESM.docx]

**Appendix 4: Allied Health Program Costs**

| **Human Resource** | **Cost** |
| --- | --- |
| 0.4 EFT Occupational Therapist Grade 2, Year 4 (6 months) (including 20% on-costs) | $32,709 |
| 0.4 EFT Physiotherapist Grade 2, Year 4 (6 months) (including 20% on-costs) | $32,709 |
|  |  |
| **Equipment** ^a^ |  |
| Fleet Vehicle x 1 | $23,679 |
| Laptop computer x 2 | $4,800 |
| Headsets with microphone x 2 | $450 |
| Mobile Phone x 2 | $2,000 |
| Aids and equipment (30 day hire cover) | $2,164 |
|  |  |
| **Software** |  |
| Videoconferencing license | $300 |
| Home exercise program license | $56 |
| **TOTAL (AUD)** | $98,867 |
| **Number of Patients** | 69 |
| **Cost per patient** | $1,432.85 |

^a^ Phoneline, wifi and HealthDirect platform costs not included in calculation as they are existing organisation-wide resources
