# Supplementary figures and images for: “The right people at the right time”: process evaluation of a novel allied health hospital in the home service for people with cancer
Source: Support Care Cancer. 2025 Jul 5;33(7):658. doi: 10.1007/s00520-025-09694-1 (PMC12228668; doi:10.1007/s00520-025-09694-1)

**Appendix 5: Community Support Referrals**


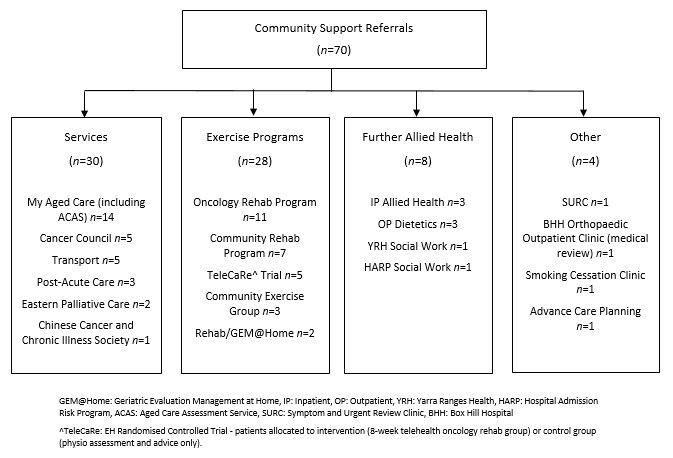

Supplement: Supplementary file 5 — (DOCX 62.1 KB) [file 520_2025_9694_MOESM5_ESM.docx]
