# Supplementary material for: “The right people at the right time”: process evaluation of a novel allied health hospital in the home service for people with cancer
Source: Support Care Cancer. 2025 Jul 5;33(7):658. doi: 10.1007/s00520-025-09694-1 (PMC12228668; doi:10.1007/s00520-025-09694-1)
Supplement: Supplementary file 6 — (DOCX 30.4 KB) [file 520_2025_9694_MOESM6_ESM.docx]

**Appendix 6: Selected Patient and Caregiver Comments Mapped to Proctor Model for Implementation**

| Proctor Outcome | Sub-theme | Quote |
| --- | --- | --- |
| Satisfaction | ***Patient First*** | - *“Your program is wonderful and helped our family a lot.”* - *“Thank you for a great program. We were so lucky to have your service.”* - *“Their patience and care are amazing and so grateful to have had them support!”* - *“I felt very supported by this program and by OT’s care, compassion and prompt follow-up of services offered. First class!!”* |
|  | ***Trust*** | - *“I am EXTREMELY grateful to both the occupational therapist and physiotherapist who attended our home - they made [patient] and my life so much better.”* |
|  | ***Safety*** | - *Suggestion: “Quarterly check in and reviews at day appointments or home to ensure progress or capture any deterioration in mobility.”* |
|  | ***Flexibility*** | - *“Was conducted in the home.”* - *“Not having to travel in my condition.”* - *“Home visits at start of journey. Availability on phone for suggestions. Awareness of support being there.”* - *“As I have been having radiation every day for 5 weeks and was very tired it was such a relief to have Hospital in the Home”* - *“Ongoing support and ability to do home visits when travel or semi covid restrictions were in place.”* - *“The service provided by Eastern Health were exemplary and they were accessible at anytime I needed them.” When they became involved to help me look after [patient] at home - it made my life so much easier!”* - *“Home visits have been a blessing at a time when it was very difficult to leave home.”* |
|  | ***Clinical Skills*** | - *“The knowledge, caring, respectful manner of allied health staff. The help with organising equipment for our loved one to remain independent at home. The follow up phone calls to see how things were going.”* - *“The physiotherapy exercises suggested for my specific case - clever practical suggestions.”* - *“Encouraging, tactful and professional.”* - *“Lots of good advice.”* - *“The exercises I was shown to do to help improve my health. Being told about the therapy group I can join with people who have the same affliction as me.”* - *“I found the occupational therapy extremely helpful in that they sourced all the things [patient] needed to enable him to remain at home when he was really ill.”* - *“Knowing I could discuss my concerns with a qualified person.”* - *“Knowledge and education and able to trial and test equipment to support day to day home life when needed.”* - *“Competent, polite, tactful staff.”* - *“All staff who came to us have been so very helpful. They gave us more than medical advice, it's their understanding of the whole situation we have and helping us connect with various services, all that are so assuring and appreciated!”* |
|  | ***Collaboration*** | - *“Little ideas they were able to give me to help my husband.”* |
|  | ***Communication*** | - *“Friendly and informative.”* - *“Having someone to talk and help and this was done in a kind, caring and helpful way.”* - *“Patience and clear communication for loved ones.”* - *“Very personal, friendly and helpful manner.”* - *“Made communication easy, as I do have some hearing loss.”* |
|  | ***Leadership*** | - *“No challenges. Everyone involved were very supportive and helpful and nothing was too much trouble for staff to follow up.”* - *“Very prompt follow up of services offered i.e. taxi vouchers, disability parking etc”* |
|  | ***Resources*** | - *“Brought equipment we either could use or examples of what we could buy.”* |
